# Structural and Non-Structural Deterioration After Biological Aortic Valve Replacement: Long-Term Outcomes of 918 High-Risk Patients

**DOI:** 10.3390/jcdd13020087

**Published:** 2026-02-11

**Authors:** Jan Hlavička, Julian Landgraf, Andreas Winter, Mascha von Zeppelin, Yasemin Ilgin, Razan Salem, Florian Hecker, Thomas Walther, Tomas Holubec

**Affiliations:** Department of Cardiovascular Surgery, Heart and Vascular Centre, University Hospital Frankfurt, Theodor-Stern-Kai 7, 60596 Frankfurt am Main, Germany; jan.hlavicka@centrum.cz (J.H.); jlandgraf1@gmail.com (J.L.); andreas.winter@unimedizin-ffm.de (A.W.); mascha.vonzeppelin@unimedizin-ffm.de (M.v.Z.); yasemin.ilgin@unimedizin-ffm.de (Y.I.); razan.salem@googlemail.com (R.S.); florianhecker@gmx.de (F.H.); thomas.walther@unimedizin-ffm.de (T.W.)

**Keywords:** aortic valve, replacement, structural change

## Abstract

Introduction: The global disease burden of aortic valve disease is already substantial and is projected to rise significantly in the coming decades. Aortic valve replacement (AVR) with a biological prosthesis has become highly popular and commonly used. This study aims to assess long-term outcomes after biological AVR with regard to structural and non-structural deterioration. Methods: In this single-centre retrospective study, 918 patients undergoing surgical AVR with a biological prosthesis at the University Hospital Frankfurt from January 2006 to July 2009 were included. The primary endpoints were freedom from reoperation and from structural and non-structural deterioration, and the secondary was long-term survival. Follow-up was completed in 95.6% with a median of 7.6 years, accounting 6610 patient-years. The mean age was 74.9 years and a median EuroSCORE II (range) was 3.34 (0.77–62.4). Twenty-two percent of surgeries were either emergent or urgent. Many patients had concomitant surgery, while coronary artery bypass grafting in 45.3% of patients was the most common. Three prosthetic valve models were used in our patient population: Carpentier Edwards Perimount (CEP) Model 2900, Model 3000 and Medtronic Mosaic (MM). Results: Reoperation occurred in 36 patients (3.9%) due to endocarditis (2.0%), aortic root aneurysm (0.1%), isolated or combined aortic stenosis or aortic regurgitation (1.9%). Freedom from reoperation at 5, 10 and 15 years was 97 ± 0.6%, 95.6 ± 0.8% and 90.3 ± 2.3%, respectively. Freedom from major stroke at 5, 10 and 15 years was 97.9 ± 0.0%, 96.4 ± 0.8%, and 96.1 ± 0.08%, and freedom from major bleeding event at 5, 10 and 15 years was 98.5 ± 0.4%, 95.7 ± 0.9% and 92.7 ± 2.2%, respectively. A subgroup analysis of the Carpentier Edwards (CEP) valves and the Medtronic Mosaic (MM) valves showed no significant differences regarding the primary endpoints. The overall survival at 5, 10 and 15 years was 67 ± 1.7%, 39.8 ± 1.8%, and 15.1 ± 2.2% respectively. The Kaplan–Meier survival estimator was 96 ± 2.2 months. Conclusion: This study showed a good long-term survival of surgical AVR with biological prostheses in relatively high-risk and elderly patient population. All biological prosthetic valves showed good long-term durability with low levels of complications and reoperations. The different models did not show any significant differences. Surgical AVR remains a valuable therapeutic option even though transcatheter aortic valve implantation has been greatly expanded since its introduction.

## 1. Introduction

Valvular heart disease is a common cause of cardiovascular morbidity and mortality [1], and a significant allocation of health resources is required to treat affected patients [2]. Due to population ageing and a lack of effective prevention strategies, the disease burden is projected to increase in the coming decades [3]. In 2017 there were an estimated 12.6 million cases of calcific aortic valve disease globally. This included calcific aortic valve (AV) disease. The age-standardised prevalence did not increase significantly between 1990 and 2017, while the number of cases rose by 124% and calcific AV disease accounted for an estimated 102,700 deaths globally in 2017 [4]. The prevalence increased significantly with age. While there is a prevalence of 0.02% in the age group 18–44, the estimated prevalence in the group over 75 years of age was 2.8% [5]. Surgical AVR (SAVR) with biological prostheses are more frequently used in younger patients [6,7]. Long-term durability of these prostheses is therefore becoming increasingly important, especially in the period of increased transaortic valve implantation (TAVI) frequency. All bioprostheses are prone to dysfunction (BVD) or failure (BVF), such as structural or non-structural deterioration. The former definition of BVF based on the need for reintervention or death underestimated its real incidence and clinical importance [8,9].

The aim of the present study was to assess the long-term outcomes after SAVR using a bioprosthesis regarding freedom from reoperation or reintervention (valve-in-valve TAVI) due to BVF, freedom from structural and non-structural BVD, and freedom from thrombo-embolic and bleeding events, as well as overall survival. Different models of bioprosthesis were evaluated on whether they may affect the incidence of BVF.

## 2. Materials and Methods

### 2.1. Ethics Statement

This study was performed in accordance with the Declaration of Helsinki (revised 2013) and approved by the local Ethics Committee of University Hospital Frankfurt (No. 20-782 from 23 June 2020). The written informed consent was waived due to the retrospective form of the study. The article will be published in accordance with the Supplementary Materials (STROBE reporting checklist).

### 2.2. Study Design and Patient’s Management

The present study is a retrospective single-centre clinical study. There were a total of 1000 patients who underwent surgical AVR with a biological prosthesis at Frankfurt University Hospital between January 2006 and July 2009. All patients who received an aortic valve replacement with a prosthesis of the following models were included in the study: Carpentier-Edwards Perimount, Carpentier-Edwards Perimount Magna, and Medtronic Mosaic.

Patients who received other prosthesis models were excluded. Finally, 918 patients were included and evaluated in the study. The 82 remaining patients received two types of self-expandable valves—ATS 3F—Enable (ATS/Medtronic, Minneapolis, MN, USA) and the first generation of Perceval (Sorin/LivaNova, Milan, Italy). All patients received transthoracic echocardiography (TTE) as well as direct coronary angiography. Relevant carotid artery sclerosis was excluded using Doppler sonography in patients older than 50 years and lung functional assessments were performed one day prior surgery. Intraoperative transoesophageal echocardiography, as a standard procedure in our department, was performed in all patients to evaluate ventricular function prior to and after cardio-pulmonary bypass (CPB), to assess exact valve pathology and prosthetic valve function after implantation. Subcutaneous low-molecular-weight heparin administration was started 6 h after surgery for the first postoperative days. Oral antiplatelet therapy with Aspirin was administered from the first postoperative day until 3 months postoperatively. Only in patients with repeated postoperative atrial fibrillation was oral anticoagulation with coumadin initiated. All patients received TTE at discharge.

The perioperative data of the patients included in the study were taken from the hospital’s internal documentation programme and the corresponding digital archive. To obtain a follow-up, a standardised questionnaire was sent by mail to all patients that were not known to have died. Subsequently, current cardiological findings with echocardiographic follow-up were requested from the treating general practitioner or cardiologist. Patients who could not be reached in this way were interviewed by telephone. The data obtained were entered into the hospital’s internal cardiac surgery database and subsequently imported into the statistical program. Follow-up (FU) was completed in 95.6% with a median duration of 91 months, counting 6610 patient-years.

Diabetes mellitus was considered present if patients were treated with insulin or oral antidiabetics. Any form of chronic obstructive pulmonary disease (COPD) was included (GOLD 1–4). Peripheral artery disease was included if it had been diagnosed before or during preoperative evaluation. Chronic kidney disease was considered present if patients had a calculated glomerular filtration (GFR) < 60 or needed dialysis. Measurements of pulmonary hypertension, if available, were obtained from echocardiographic exams or cardiac catheterisation. At least two of the following criteria must be met to identify postoperative low cardiac output syndrome (LCOS): CI < 2.0 L/min/m^2^; systolic blood pressure < 90 mmHg or a decrease > 20% compared to preoperative levels; central venous pressure > 15 mmHg; temperature gradient > 5 °C between central and peripheral sites with cold limbs; or urine output < 0.5 mL/kg/h for more than 2 h [8]. A standardised definition for the bioprosthetic valve dysfunction according to the last update of Pibarot et al. and Capodano et al. was used [9,10].

### 2.3. Statistical Analysis

Metric variables were first tested for normal distribution using the Shapiro–Wilk test. If the variable showed a normal distribution, the Levene test was employed to test for the equality of variances. In case of equal variances, Student’s *t*-test was used to calculate a *p*-value, and in case of unequal variances, Welch’s *t*-test was used. If the variable did not show a normal distribution the *p*-value was calculated using the Mann–Whitney-U test. A *p*-value < 0.05 was considered statistically significant. For the characterisation of our data sets, descriptive statistics with calculations of the mean, median and range of values in the data sets were obtained. Kaplan–Meier curves, along with mortality tables and Kaplan–Meier survival estimators, were acquired to analyse FU data and visualise the primary and secondary endpoints. Several different statistical formulae were employed to assess the comparability between the CEP and MM cohorts. For nominal and ordinal variables, Pearson’s Chi-Square test was used to calculate a *p*-value. Statistical analysis was performed using the IBM^®^ SPSS^®^ Statistics software program (version 28 for MS Windows, IBM Corporation, Armonk, NY, USA).

## 3. Results

### 3.1. Patients

The baseline patient characteristics are summarised in Table 1. Diagnosed comorbidities in our patient population included any form of diabetes mellitus, COPD (GOLD 3 + 4 3.9%), peripheral artery disease, chronic kidney disease and any grade of pulmonary hypertension (65.4% mild and severe 10.1%). Only 17 patients (1.7%) presented with acute myocardial infarction before surgery. Only 4.0% of the patients had no limitation of physical activity according to the New York Heart Association (NYHA I), 53.2% of them were symptomatic in the meaning of NYHA III, and heart failure at rest (NYHA IV) was present in 6.5% of patients. While only 1.6% of patients had angina at rest according to the Canadian Cardiovascular Society (CCS) angina classification (CCS IV), 24.8% stated that they did not experience any angina pectoris. Six of them (0.7%) experienced the transaortic balloon valvuloplasty before surgery.

The most common aetiology of aortic valve disease in our study population was degenerative calcific AV stenosis, with 82.9%. Bicuspid AVs were found in 12.2% of patients. Isolated AV regurgitation was the indication for surgery in only in 6.9%, in the combination with stenosis occurring insufficiently in 13.9%.

### 3.2. Intraoperative Data

The intraoperative variables are summarised in Table 2. Coronary artery bypass grafting (CABG) was the most frequent concomitant procedure performed along SAVR in our study population. In 7.5% of cases an additional heart valve procedure was performed: mitral valve repair in 2.3%, mitral valve replacement in 1.6%, and tricuspid valve repair in 3.8%, and in 5.1% the surgeons operated on three heart valves during the same surgery. Additional replacement of the ascending aorta was performed in 3.1% of our cases.

There were three models of prosthetic valves used for SAVR in the study population. The Carpentier-Edwards Perimount and Perimount Magna valve (Edwards Lifesciences Corporation, Irvine, CA, USA) were used in most patients. The two models were in the following comparison included in the same group against Mosaic. The valve sizes 21 mm, 23 mm and 25 mm were used most frequently (in 33%, 36% and 20.9% resp.). Other sizes accounted for only 9.2% of cases.

### 3.3. Early Postoperative Results and Follow-Up Data

**Figure 1:**
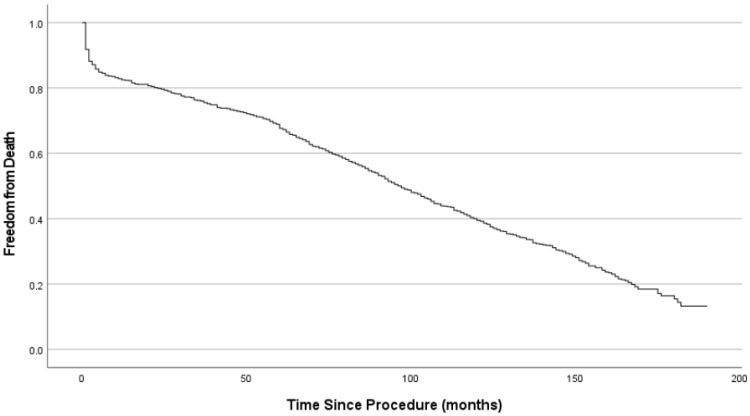
Kaplan–Meier curve showing the freedom from all-cause death.

**Figure 2:**
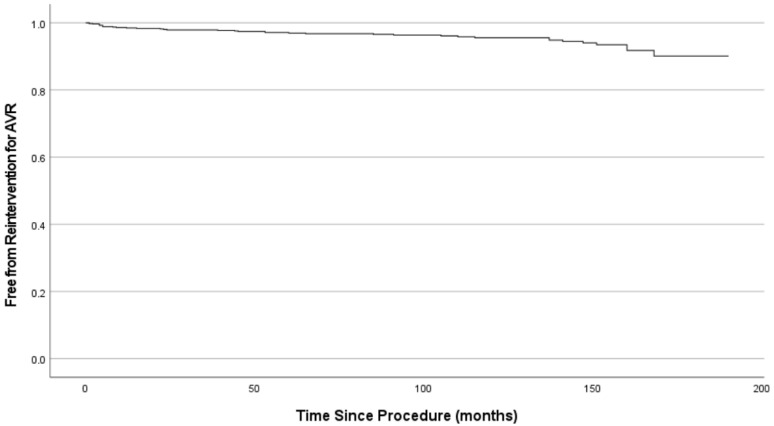
Freedom from reintervention for AVR.

Early postoperative data are detailed in Table 3. In general, inotropic agents were used for less than 12 h in the majority (66.2%) of patients. The incidence of any form of stroke was low. Cause of mortality included leading any form of infection (33.3%), multiorgan failure (25.8%) and myocardial ischemia (25.0%). The multivariable Cox regression risk analysis has identified following factors, negatively impacting early mortality: age (HR = 1.07, 95% CI: 1.02–1.13, *p* = 0.012), cardiac decompensation before surgery (HR = 1.37, 95% CI: 1.01–1.72, *p* = 0.006), rethoracotomy (HR = 2.01, 95% CI: 1.01–4.05, *p* = 0.029), postoperative stroke (HR = 1.47, 95% CI: 1.07–2.02, *p* = 0.016) and low cardiac output syndrome (HR = 1.75, 95% CI: 1.15–2.67, *p* = 0.010). At the time of the latest FU, 676 patients died. Two-hundred-two alive patients could be reached to obtain detailed data on their medical history and current condition. Forty (4.4%) patients were lost to FU. All complications mentioned in the FU occurred after the initial hospitalisation (Table 4). In 1.5% of patients, echocardiographic examinations showed a dysfunctional prosthetic valve, but due to a high surgical risk or refusal by the patient, no surgical or interventional procedure was performed. Early mortality during or after the procedure results in a sharp initial drop of the curve. Survival at 5, 10 and 15 years was 67 ± 2%, 39 ± 2%, and 14 ± 2% respectively (see Figure 1). The Kaplan–Meier survival estimator was 96 ± 2.2 months (≈8 years). Figure 2 shows a Kaplan–Meier curve plotting the freedom of reintervention for AVR against the time since the index procedure. The graph shows an increase in interventions close to the end of the FU period.

### 3.4. Comparison of CEP and MM

For the comparison of the CEP and MM prosthetic valves, all relevant patient characteristics were examined to assess the comparability of the two study cohorts. Preoperative baseline characteristics were similar for both groups with three exceptions: patients receiving CEP had diabetes less often (28% vs. 38%; *p* = 0.020), a higher grade of dyspnoea (NYHA III + IV 57% vs. 70%; *p* = 0.014) and historical myocardial infarction (1% vs. 4%; *p* = 0.020). Generally, the patients with MM had more comorbidities, but the CEP patients received more acute and complex surgeries, such as additional heart valve procedures, ligation of the left atrium appendage, ablation, subvalvular myectomy and surgery on the ascending aorta. This fact resulted in the significantly longer aortic cross-clamp time as well as the mean cardio-pulmonary bypass time. On the other hand, there were no significant differences in the surgical approach used, the frequency of concomitant CABG, and the size of the prosthetic valve (*p* = 0.107). Early postoperative data are detailed in Table 5, and only show a longer stay on the intensive care unit (ICU) in CEP patients (median [range]: 5.38 [0–139] vs. 4.38 [1–48]) in days. Other important markers of early postoperative outcome such an early—in hospital and 30-day—mortality were also non-significant. The duration of mechanical ventilation was comparable between groups.

Table 6 details the FU data collected for the CEP and MM cohorts. The *p*-values obtained using the Pearson Chi-Square test showed no significant differences between the two valve groups.

**Figure 3:**
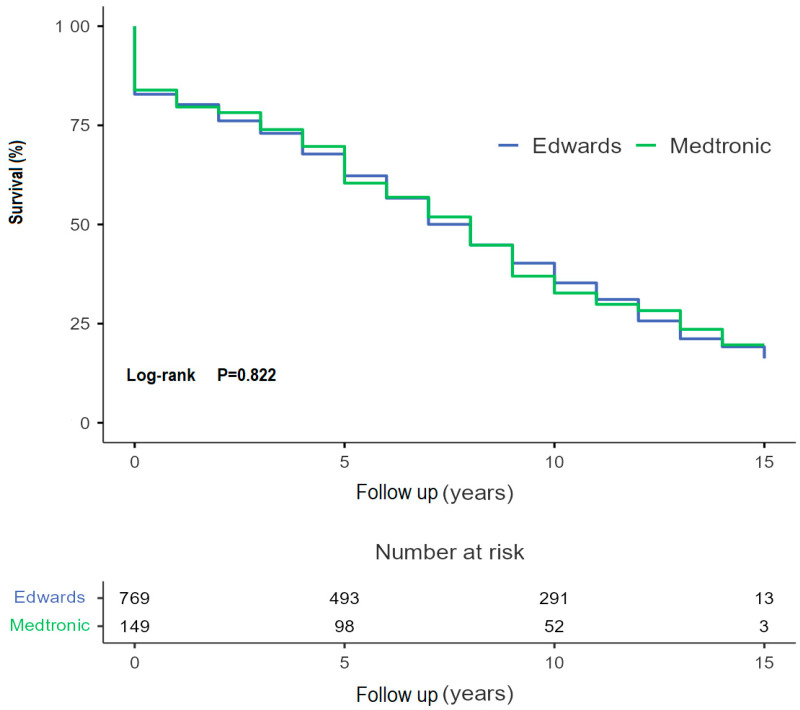
Freedom from all-cause death.

**Figure 4:**
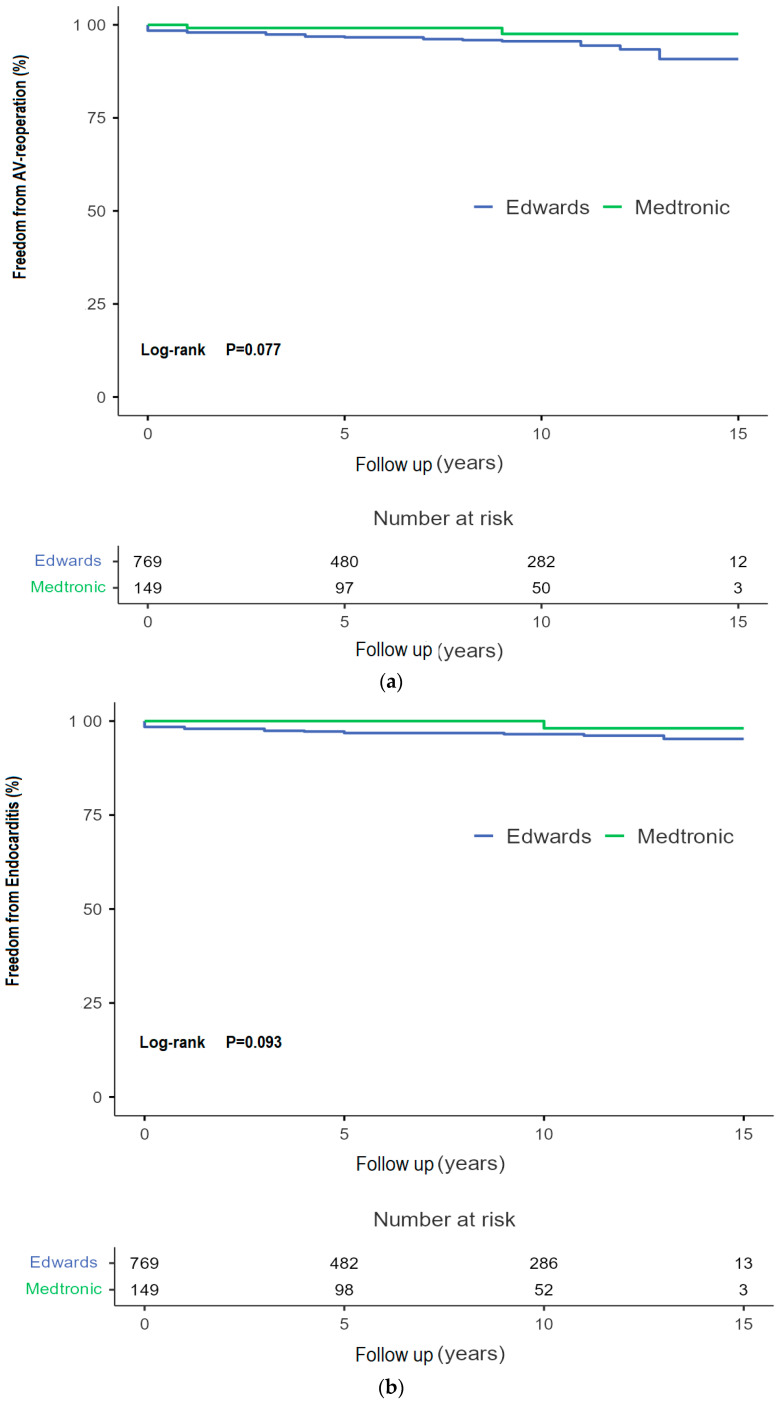
(**a**) Freedom from AV-reintervention. (**b**) Freedom from endocarditis.

**Figure 5:**
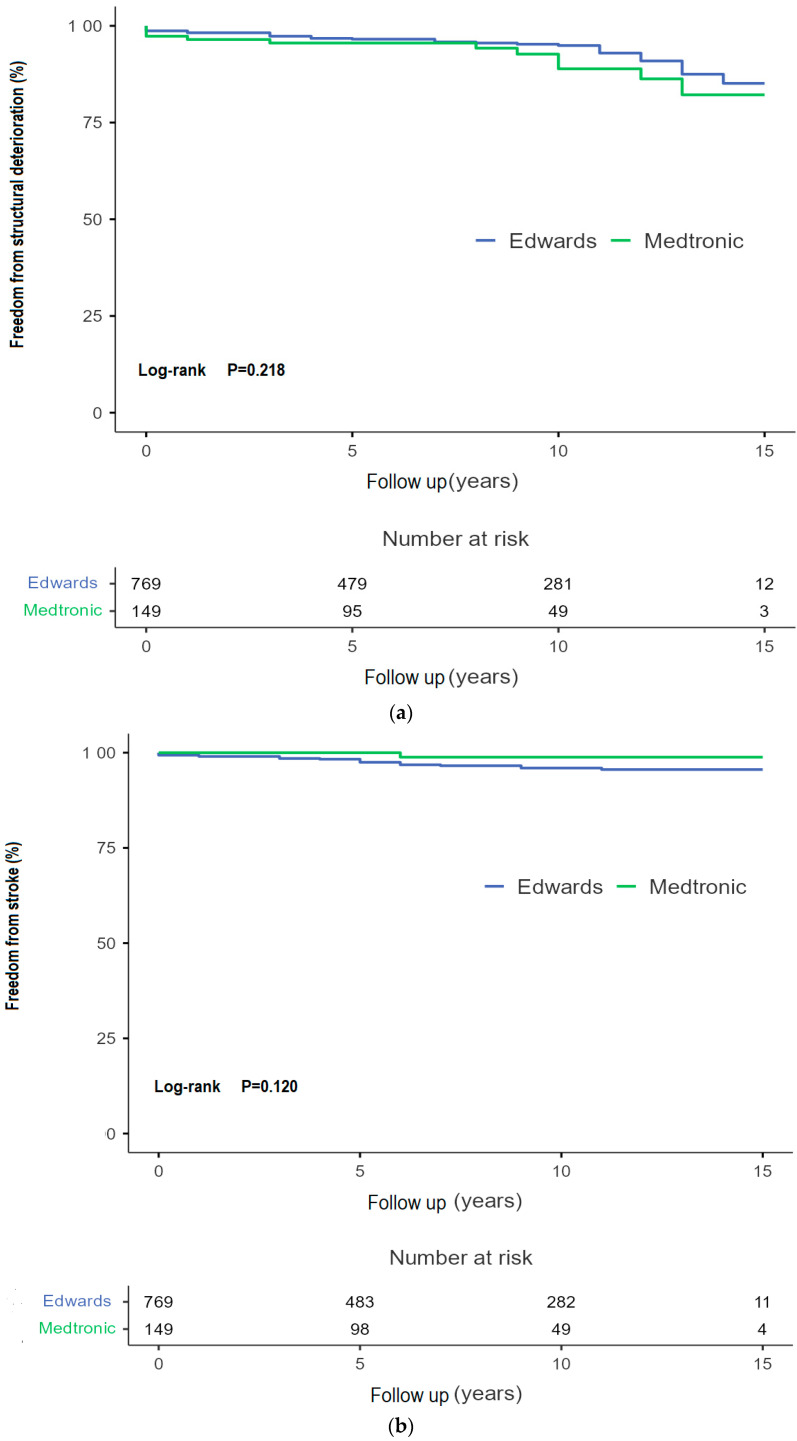
(**a**) Freedom from structural deteriorations. (**b**) Freedom from any stroke.

The Kaplan–Meier survival estimate for patients from the CPE group was 96.06 [91.32–100.80] months versus an estimated 96.20 [85.64–106.76] months for the MM group (Figure 3). There was no significant difference in incidence of valve dysfunction without reoperation between the two models (*p* = 0.680). A decline can be seen after 120 months (10 years) in both the CEP and MM cohort. The Kaplan–Meier estimate for the CEP group is 185.65 months versus 180.47 months in the MM group. Figure 4a shows the freedom from reintervention for AVR. No significant differences between the groups have been proven. The Kaplan–Meier estimate for freedom from reoperation was 179.78 [176.74–182.80) months for the CEP groups versus 181.33 [177.64–185.00] months for the MM group. In Figure 4b there is an estimated freedom from endocarditis of 182.29 [179.64–184.93] months for the CEP group and 182.85 [180.62–185.08] months for the MM group. Kaplan–Meier analyses were also conducted for freedom from major bleeding events (*p*-value: 0.20) and thrombo-embolic cerebral complications (*p*-value 0.49), with no significant difference from the long-term point of view. See Figure 5.

## 4. Discussion

Our single-centre retrospective study included, in total, 918 high-risk patients undergoing SAVR with two types of a biological prosthesis. The patients receiving two types of self-expandable valves—ATS 3F Enable (ATS/Medtronic, Minneapolis, MN, USA) and the first generation of the Perceval (Sorin/LivaNova, Milan, Italy) valve—were excluded because of differing design and the specific sutureless surgical technique of the implantation. The lack of mid- and long-term experiences with the self-expanding valves at that time led to their exclusion from the data set. Intrahospital mortality was 3.2%. Mortality between discharge and the day 30 was 7.0%, so the 30-day mortality resulted in 10.0%. This is higher than in many other studies examining outcomes after SAVR [11,12,13,14,15,16,17]. However, several of these studies excluded patients receiving any type of concomitant procedure leading to a lower surgical risk and hence a reduced early mortality [11,12,13,14]. Others did not include multi-valve procedures [15]. Besides this, the patients who died within the first 30 days after the index procedure showed a significantly (*p* < 0.01) higher median EuroSCORE II of 7.16 when compared to the rest of our patient cohort, who had a median EuroSCORE II of 3.22. Multi-valve procedures were also more common in this subgroup, with more than 26% of patients receiving one or two additional valve procedures beside the SAVR, compared to less than 10% in the rest of the patient cohort. This shows the increase in mortality due to individual high-risk patients in our study.

The survival rates after surgery are dependent on several factors. Age has been shown as one of the strongest factors affecting mortality, which confirmed also our multivariable Cox regression risk analysis (HR = 1.07, 95% CI: 1.02–1.13, *p* = 0.012). The mean age of our patient population was 74.9 [47–93] years. The survival rate at 5, 10 and 15 years was 67 ± 2%, 39 ± 2%, and 14 ± 2% respectively. The Kaplan–Meier survival estimator was 96 ± 2.2 months (≈8 years). A systematic review of 93 studies on the outcomes of SAVR found survival rates of 78.4% at 5 years, 57.0% at 10 years and 39.7% at 15 years. A subgroup analysis of patients aged 65 to 75 years showed a survival rate of 81.4.% after 5 years with a median survival estimate of 12 years after surgery, while patients aged 75 to 85 years (our group) had a rate of 67.4% with a survival estimate of 7 years [18]. The life expectancy of 75-year-old residents of Germany between the years 2006 and 2008 varies between 10.34 years for men and 12.38 years for women [19]. The Kaplan–Meier estimate for freedom from all-cause death in our patient population with a median age of 75 was 8 years. As Maeda et al. have proven, patients with intermediate risk have, after 5 years following SAVR, 7% lower survival compared to the general population (77% vs. 84%, *p* = 0.03) [20]. Another study of Hernandez-Haquero et al. found a fully restored life expectancy in patients after SAVR with similar survival to the general population. There were, however, significant differences between the patient population of that study and ours. Among other things, the patients with concomitant procedures other than CABG were excluded [11].

Several studies have shown the good long-term durability of biological aortic valve prostheses. The study of Bourguignon et al. estimated the durability of the CEP valve to be as high as 19.7 years [21]. Reintervention for AVR occurred in 3.9% of patients in our study population, and 4.4% in the CEP group, which corroborates the results of Bourguignon et al., with 98.1% freedom of redo in the age group > 70. Rates of reintervention/reoperation in other studies vary from 0.79% in the 15 years FU [22] to 17.63% in 20 years FU [23]. The high rate of reoperation in the latter study of Bourguignon et al. was shown in a cohort of patients aged 50 to 65 years. The rates of another study by Biglioli et al., with similar patient characteristics to our patient population, did not exceed 8.56% [16]. In our retrospective analysis the causes for reoperation were mostly structural valve deterioration, though endocarditis was also a frequent indication for reintervention/reoperation. This was consistent with some other studies, providing the reasons for reoperation [14,21,24]. During FU there were 31 cases of thrombo-embolic cerebral complications. In total, 3.4% of patients suffered either a minor or major stroke [22,25,26,27]. Other studies of long-term outcomes after SAVR reported rates of stroke between 4.17% and 10.92%. High rates of combined procedures and concomitant aortic surgery should be associated with higher risk of stroke in our cohort. Early postoperative bleeding complications can lead to a substantially increased mortality and impact the length of stay during the initial hospitalisation [28]. The relatively high incidence of early major bleeding events resulting in re-exploration (13.8%) in our patient population may be the result of a high-risk population with inclusion of multi-valve procedures (12.6%), endocarditis (1.9%), reoperations (6.3%) and complex aortic surgery (11.3%) in our study. We identified 25 cases of major bleeding during FU, amounting to 2.72% of patients. Although biological aortic valve prostheses do not require lifelong anticoagulation and therefore have a lower risk of late major bleeding events [29], other studies of long-term outcomes after SAVR reported rates of major bleeding in FU between 0.39% and 2.33% [15,17,26]. The three prosthetic valves employed in our study were the CEP valve, the CEP Magna valve and the MM valve. CEP Magna (Model 3000) has been used in 1.5% cases only. We fused it into the large group of CEP (Model 2900 and 3000), at 83.7%, to prevent the fragmentation of the data. A comparison between the CEP valves and MM valve found no significant difference for our primary and secondary endpoints. Though the trend of lower incidence of reoperation for AVR (*p* = 0.08) and endocarditis (*p* = 0.09) in the MM group is obvious, it can generally be said that the Medtronic Mosaic valve was more often used for a planned, isolated AVR than the opposite model, which is the explanation for the lower incidence of structural and non-structural failure of the prosthesis. Other studies comparing the CEP valve and MM valve also found no significant differences in long-term survival, suggesting that the valves are equally valid choices for AVR [16]. One study reported higher transvalvular gradients in patients that had received the MM valve in FU echocardiography without clinal consequence [12]. Another study also found higher transvalvular gradients in the MM valve when compared to the CEP valve, but during the 5-year FU the CEP valve also showed superior survival rates [30].

Since the introduction of the TAVI procedure, there have been many studies examining early outcomes and comparing them with SAVR [31]. While the TAVI procedure was initially reserved for patients that could not undergo surgery due to high surgical risk, the indications have since been expanded [32]. The use of TAVI in younger and healthier patients with longer life expectancy makes the examination of durability of the prosthetic valves even more important [33]. There are a few studies assessing very long-term outcomes of 10 years or more after TAVI. Blackman et al. examined the durability of transcatheter aortic valve prostheses and reported a very low incidence of structural degeneration in an FU of up to 10 years [32]. A late echocardiographic FU rate of only 15.8% makes the results very uncompleted. Thyregod et al. presented a randomised NOTION study comparing SAVR and TAVR among low-risk patients with no difference in risk of major clinical outcomes in 10 years after treatment. The risk of severe bioprosthesis structural valve deterioration (SVD) was lower after TAVR compared with SAVR, while the risk of BVF was similar. Although, reference to the NOTION trial, with respect to durability, should be used cautiously. As Thyregod et al. disclosed, “the surgical bioprostheses with externally mounted leaflets on the stent and now known decreased durability was used, and only 10% of SAVR patients received a pericardial bioprosthesis which has demonstrated improved durability compared with porcine bioprostheses” [34]. A systematic review of studies with an FU upwards of 5 years showed an aggregated survival rate of 48% at 5 years and 28% at 7 years [35]. Sathananthan et al. reported survival rates of 37% at 5 years and 8.4% at 10 years in a high-risk and elderly patient population [36]. Erlebach et al. reported survival rates of 44.4% at 5 years and 10.3% at 10 years in a slightly younger, but high-risk, patient cohort [37]. As the studies mentioned above only included elderly and high-risk patients, a direct comparison of their results to our outcomes is not useful. According to a study by Stähli et al., our patient population would qualify as an intermediate risk cohort with a mean EuroSCORE II of 5.59 [38]. Makkar et al. used the STS risk score to evaluate the surgical risk of patients requiring aortic valve replacement and only recruited intermediate risk patients in the PARTNER 2 trial. The survival rate at 5 years in the TAVI group was 54%, compared to 67% of our patient population. This difference might be explained by a higher mean age of the TAVI patients (81.5 vs. 75.0 years) or the inclusion criteria of the PARTNER 2 trial (only patients with severe, symptomatic aortic stenosis). The low incidence of the permanent pacemaker implantation after SAVR in our study (2.2%) demonstrates clearly the surgical method being superior to the TAVR (18.9%), as the meta-analysis of Ullah et al. has shown [39].

## 5. Conclusions

In summary, SAVR remains a viable treatment for aortic valve pathologies with low rates of reoperation, excellent long-term durability of the employed valve prostheses and good long-term outcomes even in a higher-risk elderly patient population. A comparison of the different models—Carpentier Edwards Perimount and Medtronic Mosaic—showed no significant differences in postoperative outcomes.

## 6. Limitations and Strengths of the Study

The retrospective nature of this study allowed for the inclusion of a high number of patients but introduces the inherent flaws of this study design. The new generation of biological valve prostheses cannot be automatically compared with the results of our study. Although it is to be expected that new technologies of valve preservation and materials used will enable even better outcomes, long-term follow-up cannot exclude many potential biases, entering between exposition and outcome. But any retrospective study can predict the future developments or understand unexplored areas based on existing trends. On the other hand, the large cohort of patients included represents a robust basis for high-quality data interpretation.

## Figures and Tables

**Table 1:** Baseline patient characteristics.

Variables	*n* = 918
Age, mean ± SD	75.1 ± 6.9
BMI median	26.8 (18–41)
EuroSCORE II, median (range)	3.34 (0.77–62.4)
Female	397 (43.2%)
NYHA > II	544 (60%)
Moderate PH	600 (65.4%)
Severe PH	93 (10.1%)
SR	738 (80.4%)
AFib	180 (19.6%)
Redo	162 (17.7%)
Redo AVR	10 (1.1%)
EF > 55%	575 (63.1%)
EF 45–55%	144 (15.8%)
EF 30–45%	163 (17.9%)
EF < 30%	29 (3.2%)
CHD	407 (37.5%)
AH	771 (89.3%)
DM	276 (30.7%)
COPD	212 (25.9%)
CKD	325 (32.4%)
Stroke	71 (7.8%)
PAD	102 (11.9%)
Al > 2	186 (20.3%)

Data are presented as mean ± SD, median (range), where appropriate, or as numbers (%). BMI—body mass index, NYHA—New York Heart Association functional class, PH—pulmonary hypertension, HR—heart rhythm, SR—sinus rhythm, AFib—atrial fibrillation, AVR—aortic valve replacement, EF—left ventricle ejection fraction, CHD—coronary heart disease, AH—arterial hypertension, DM—diabetes mellitus, COPD—chronic obstructive pulmonary disease, CKD—chronic kidney disease, PAD—peripheral artery disease, AI—aortic insufficiency.

**Table 2:** Intraoperative variables.

Variables	*n* = 918
Elective	720 (78.4%)
Urgent	170 (18.5%)
Emergent	28 (3.1%)
Aortic cross-clamp time in minutes (range)	79 (29–247)
CPB time in minutes (range)	115 (46–468)
Full sternotomy	581 (63.3%)
Minimal invasive surgery	337 (36.7%)
Concomitant ACB	416 (45.3)
1 additional valve OP	69 (7.5%)
2 additional valve OP	47 (5.1%)
LAA Ligation	256 (27.9%)
LA Ablation	20 (2.2%)
Myectomy	130 (14.2%)
Surgery of ascending aorta	104 (11.3%)
CE Perimount (Model 2900)	755 (82.2%)
CE Perimount Magna (Model 3000)	14 (1.5%)
Medtronic Mosaic	149 (16.2%)

Min—minutes, CPB—cardio-pulmonary bypass, OP—operation, LAA—left atrium appendage, LA—left atrium, CE—Carpentier Edwards.

**Table 3:** Early postoperative data.

Variables	*n* = 918
Inotropic Agents > 12 h	310 (33.8%)
Mechanical Ventilation (h) Median Mean [Range]	12.0 80.1 [3–3199]
Time at ICU (d) Median Mean [Range]	2.0 5.2 [0–139]
Wound Healing Disorder Sternum Superficial Deep Operative Revision	113 (12.3%) 57 (6.2%) 15 (1.6%) 41 (4.5%)
Re-exploration due to bleeding/tamponade	127 (13.8%)
Postoperative MI	1 (0.1%)
Gastrointestinal Complications Bleeding Ischemia Perforation	43 (4.7%) 32 (3.5%) 9 (1.0%) 2 (0.2%)
Cerebral Accident (stroke) Minor Major Coma	18 (2.0%) 5 (0.5%) 7 (0.8%) 6 (0.7%)
Reintubation	82 (8.9%)
Tracheotomy	79 (8.6%)
Renal Replacement Therapy	141 (15.4%)
Postoperative Implantation Pacemaker	20 (2.2%)
Low Cardiac Output syndrome Medical IABP ECMO	65 (7.1%) 42 (4.6%) 12 (1.3%) 11 (1.2%)
Early Mortality In Hospital 30 days	93 (10.2%) 29 (3.2%) 64 (7.0%)

h—hours, ICU—intensive care unit, d—days, MI—myocardial infarction, IABP—intra-aortic balloon pump, ECMO—extracorporal membrane oxygenation.

**Table 4:** Follow-up data.

Variables	*n* = 878
Dysfunction Without Reoperation	14 (1.5%)
Reoperation	36 (3.9%)
Cause of Valve Dysfunction	
Stenosis	16 (32%)
Regurgitation	8 (16%)
Combined Disease	11 (22%)
Vegetation/Perforation	14 (28%)
Unknown	1 (2%)
Cerebral Complications	31 (3.4%)
Minor	8 (0.9%)
Major	23 (2.5%)
Major Bleeding Event	25 (2.8%)
GIT	18 (2.0%)
Intracranial	7 (0.8%)
Endocarditis	25 (2.7%)
Late Death	
Alive at time of FU	202 (22.0%)
Dead	676 (73.6%)
Lost to FU	40 (4.4%)

GIT—gastrointestinal tract, FU—follow-up.

**Table 5:** Postoperative results.

Variables	CEP *n* = 769	MM *n* = 149	*p*-Value
Median time of mechanical ventilation in hours [SD]	12 [243.5]	12 [162.8]	0.288
Median time in ICU in days [SD]	2 [11.0]	2 [7.6]	0.054
Re-exploration due to bleeding	112 (14%)	15 (10%)	0.156
Postoperative MI	1 (0.1%)	0	1.000
Gastrointestinal Complications	35 (4.6%)	8 (5.4%)	0.505
Reintubation	71 (9.2%)	11 (7.4%)	0.453
Tracheotomy	66 (8.6%)	13 (8.7%)	0.987
Renal Replacement Therapy	122 (15.9%)	19 (12.8%)	0.386
Pacemaker Implantation	25 (3.3%)	8 (5.4%)	0.226
Low Cardiac Output			0.396
Medical Management	33 (4.3%)	9 (6%)	
IABP	10 (1.3%)	2 (1.3%)	
ECMO	11 (1.4%)	0	
Early Mortality			0.593
In Hospital	26 (3.4%)	3 (2%)	
30 days	55 (7.2%)	9 (6%)	

CEP—Carpentier Edwards Perimount Model 2900 or Model 3000, MM—Medtronic Mosaic, SD—standard deviation, ICU—intensive care unit, MI—myocardial infarction, IABP—intra-aortic balloon pump, ECMO—extracorporeal membrane oxygenation.

**Table 6:** Follow-up data CEP vs. MM.

Variables	CEP *n* = 769	MM *n* = 149	*p*-Value
Dysfunction without reoperation	11 (1.4%)	3 (2%)	0.485
Reoperation	34 (4.42%)	2 (1.3%)	0.103
Embolic event	31 (4%)	4 (2.7%)	0.639
Major Stroke	20 (2.6%)	3 (2%)	0.902
Major Bleeding Event	18 (2.3%)	7 (4.7%)	0.464
Endocarditis	24 (3.1%)	1 (0.7%)	0.104
Late Death	568 (74%)	108 (72.5%)	0.936
Endocarditis	11 (1.4%)	3 (2%)	0.485

CEP—Carpentier Edwards Perimount Model 2900 or Model 3000, MM—Medtronic Mosaic.

## Data Availability

The original contributions presented in this study are included in the article/Supplementary Materials. Further inquiries can be directed to the corresponding author.

## Supplementary Materials

The following supporting information can be downloaded at https://www.mdpi.com/article/10.3390/jcdd13020087/s1, STROBE reporting checklist.
