# Supplementary material for: Structural and Non-Structural Deterioration After Biological Aortic Valve Replacement: Long-Term Outcomes of 918 High-Risk Patients
Source: J Cardiovasc Dev Dis. 2026 Feb 11;13(2):87. doi: 10.3390/jcdd13020087 (PMC12942293; doi:10.3390/jcdd13020087)
Supplement: Supplementary file 1 [file jcdd-13-00087-s001.zip › jcdd-4085814-supplementary.pdf]

STROBE Statement—Checklist of items that should be included in reports of ***cohort studies***

	Item No	Recommendation
**Title and abstract**	1	Line 2-4: Structural and non-structural deterioration after biological aortic valve replacement: long-term outcomes of 918 high-risk patients
		Line 15-44
**Introduction**		
Background/rationale	2	Line 45-66
Objectives	3	Line 56-61 and 62-66
**Methods**		
Study design	4	Line 74-117
Setting	5	Line 96-104
Participants	6	Line 96-100
Variables	7	Line 105-117
Data sources/ measurement	8*	Line 96-104
Bias	9	Line 82-84
Study size	10	Line 75-76 and 81-82
Quantitative variables	11	Line 119-132
Statistical methods	12	Line 119-132
**Results**		
Participants	13*	Line 81-84
		Line 82-84
Descriptive data	14*	(a) Give characteristics of study participants (eg demographic, clinical, social) and information on exposures and potential confounders<br>Line 135-157
		(b) Indicate number of participants with missing data for each variable of interest<br>Line 185
		(c) Summarise follow-up time (eg, average and total amount)<br>Line 103-104
Outcome data	15*	Report numbers of outcome events or summary measures over time<br>Line 175-212
Main results	16	(*a*) Give unadjusted estimates and, if applicable, confounder-adjusted estimates and their precision (eg, 95% confidence interval). Make clear which confounders were adjusted for and why they were included<br>Line 178-183
		(*b*) Report category boundaries when continuous variables were categorized<br>Line 214-229
Other analyses	17	Report other analyses done—eg analyses of subgroups and interactions, and sensitivity analyses<br>Line 249-262
**Discussion**		
Key results	18	Summarise key results with reference to study objectives<br>Line 316-356

Limitations	19	Discuss limitations of the study, taking into account sources of potential bias or imprecision. Discuss both direction and magnitude of any potential bias<br>Line 397-406
Interpretation	20	Give a cautious overall interpretation of results considering objectives, limitations, multiplicity of analyses, results from similar studies, and other relevant evidence<br>Line 357-390
Generalisability	21	Discuss the generalisability (external validity) of the study results<br>Line 379-390
**Other information**		
Funding	22	Give the source of funding and the role of the funders for the present study and, if applicable, for the original study on which the present article is based<br>Line 411

*Give information separately for exposed and unexposed groups.

Note: An Explanation and Elaboration article discusses each checklist item and gives methodological background and published examples of transparent reporting. The STROBE checklist is best used in conjunction with this article (freely available on the Web sites of PLoS Medicine at http://www.plosmedicine.org/, Annals of Internal Medicine at http://www.annals.org/, and Epidemiology at http://www.epidem.com/). Information on the STROBE Initiative is available at http://www.strobe-statement.org.
